# Oral Ultramicronized Palmitoylethanolamide: Plasma and Tissue Levels and Spinal Anti-hyperalgesic Effect

**DOI:** 10.3389/fphar.2018.00249

**Published:** 2018-03-20

**Authors:** Stefania Petrosino, Marika Cordaro, Roberta Verde, Aniello Schiano Moriello, Gabriele Marcolongo, Carlo Schievano, Rosalba Siracusa, Fabiana Piscitelli, Alessio F. Peritore, Rosalia Crupi, Daniela Impellizzeri, Emanuela Esposito, Salvatore Cuzzocrea, Vincenzo Di Marzo

**Affiliations:** ^1^Endocannabinoid Research Group, Institute of Biomolecular Chemistry, CNR, Napoli, Italy; ^2^Epitech Group SpA, Padova, Italy; ^3^Department of Chemical, Biological, Pharmaceutical and Environmental Science University of Messina, Messina, Italy; ^4^Innovative Statistical Research SRL, Padova, Italy

**Keywords:** absorption, hyperalgesia, inflammation, micronization, palmitoylethanolamide

## Abstract

Palmitoylethanolamide (PEA) is a pleiotropic lipid mediator with established anti-inflammatory and anti-hyperalgesic activity. Ultramicronized PEA (PEA-um) has superior oral efficacy compared to naïve (non-micronized) PEA. The aim of the present study was two-fold: (1) to evaluate whether oral PEA-um has greater absorbability compared to naïve PEA, and its ability to reach peripheral and central tissues under healthy and local inflammatory conditions (carrageenan paw edema); (2) to better characterize the molecular pathways involved in PEA-um action, particularly at the spinal level. Rats were dosed with 30 mg/kg of [^13^C]_4_-PEA-um or naïve [^13^C]_4_-PEA by oral gavage, and [^13^C]_4_-PEA levels quantified, as a function of time, by liquid chromatography/atmospheric pressure chemical ionization/mass spectrometry. Overall plasma levels were higher in both healthy and carrageenan-injected rats administered [^13^C]_4_-PEA-um as compared to those receiving naïve [^13^C]_4_-PEA, indicating the greater absorbability of PEA-um. Furthermore, carrageenan injection markedly favored an increase in levels of [^13^C]_4_-PEA in plasma, paw and spinal cord. Oral treatment of carrageenan-injected rats with PEA-um (10 mg/kg) confirmed beneficial peripheral effects on paw inflammation, thermal hyperalgesia and tissue damage. Notably, PEA-um down-regulated distinct spinal inflammatory and oxidative pathways. These last findings instruct on spinal mechanisms involved in the anti-hyperalgesic effect of PEA-um in inflammatory pain.

## Introduction

Palmitoylethanolamide (PEA) is an endogenous fatty acid amide signaling molecule synthesized “*on demand*” in response to tissue injury/stress, as part of a mechanism to restore/maintain homeostasis with anti-inflammatory, pain-relieving and neuroprotective actions (Solorzano et al., 2009; Skaper and Facci, 2012; Piomelli and Sasso, 2014; Petrosino and Di Marzo, 2017). This view is supported by studies showing that PEA levels change in settings of tissue injury, especially in situations associated with inflammatory and neurodegenerative processes (Franklin et al., 2003; Petrosino et al., 2007, 2010; Bisogno et al., 2008; Loría et al., 2008; Garcia-Ovejero et al., 2009; Iannotti et al., 2016; Petrosino and Di Marzo, 2017). This hypothesis is supported by a large body of evidence showing that the systemic administration of PEA elicits anti-inflammatory, antinociceptive, and neuroprotective effects, both *in vivo* and *in vitro* (Mazzari et al., 1996; Costa et al., 2008; Genovese et al., 2008; Esposito et al., 2011; D'Agostino et al., 2012; Esposito and Cuzzocrea, 2013; Abramo et al., 2017; Skaper, 2017; Scuderi et al., 2018), as well as in man (Truini et al., 2011; Gatti et al., 2012; Marini et al., 2012; Paladini et al., 2016; Artukoglu et al., 2017; Passavanti et al., 2017; Chirchiglia et al., 2018) and companion animals (Scarampella et al., 2001; Noli et al., 2015).

The lipophilic nature of PEA presents a major challenge in its therapeutic use. PEA is practically insoluble in water and poorly soluble in most other aqueous solvents, with the logarithm of its partition coefficient (log P) being >5 (Lambert et al., 2001). Absorption of orally administered PEA is thus likely be dissolution-rate-limited, with the amount absorbed conceivably showing an inverse relation to particle size (Takano et al., 2008). Micronization is frequently applied to reduce particle size and improve the bioavailability and efficacy of very low water-soluble molecules by increasing their dissolution rate (Joshi, 2011; Leleux and Williams, 2014; Campardelli et al., 2017). Micronized pharmaceutical grade formulations of PEA obtained by jet milling (particle size distribution: 0.8–10 μm; Impellizzeri et al., 2014; Skaper et al., 2014) are currently used in human and veterinary medicine for inflammatory, hyperalgesic and allergic disorders (Petrosino and Di Marzo, 2017). Marketed PEA formulations contain: (i) unprocessed PEA (frequently referred to as naïve PEA or pure PEA; from 100 μm up to 2,000 μm); (ii) micronized PEA (PEA-m; 2–10 μm range); and (iii) ultramicronized PEA (PEA-um; 0.8–6 μm range). In the carrageenan (CAR)-induced model of rat paw inflammation, orally administered PEA-m/PEA-um markedly reduced both paw edema and thermal hyperalgesia in comparison to naïve PEA (Impellizzeri et al., 2014). PEA-m/PEA-um has a favorable safety profile in genetox assays as well as in acute and repeat dose oral toxicity studies (Nestmann, 2016).

Few pharmacokinetic studies have been reported for PEA [reviewed in Petrosino and Di Marzo, 2017] although some estimates have been attempted (Gabrielsson et al., 2016). Such studies might be complicated by issues concerning: (i) PEA natural occurrence and its synthetic/degradative machinery; (ii) multiple mechanisms of action, both direct and indirect (Smart et al., 2002; Ho et al., 2008; Petrosino et al., 2016; Petrosino and Di Marzo, 2017). The first point can compromise obtaining reliable pharmacokinetic data, since exogenous PEA—even labeled—could re-arrange with the endogenous pool of PEA through enzymatic pathways. Indeed, PEA is easily hydrolyzed by fatty acid amide hydrolase and *N*-acylethanolamine acid amidase (Cravatt et al., 1996; Ueda et al., 2001). The second point is more difficult to assess, since PEA levels in blood and tissues could be independent from its pharmacological effect. Conceivably, the latter may be the product of a cascade of events triggered by PEA but ultimately expressed through second- or third-order pathways. This concept is illustrated by the so-called “entourage effect,” a mechanism by which PEA actions result from increased levels or receptor affinity of endogenous protective compounds, such as the endocannabinoids arachidonoyl-ethanolamide (Smart et al., 2002; Ho et al., 2008; Petrosino and Di Marzo, 2017) and 2-arachidonoyl-glycerol (Petrosino et al., 2016).

With the above considerations in mind, the present study was carried out to investigate whether micronization enhances absorption of orally administered PEA in the healthy organism. Since PEA-based products are indicated for inflammatory and hyperalgesic conditions, plasma levels of PEA after oral administration of PEA-um were also measured in a rat model of acute inflammation (CAR-induced paw edema). Finally, the levels of PEA in paw, spinal cord and brain and possible correlation to underlying molecular mechanisms in the CAR model were investigated.

## Materials and methods

### Materials

Unless otherwise specified, all compounds used in this study were purchased from Sigma-Aldrich (Milano, Italy). [^13^C]_4_-PEA-um and naïve [^13^C]_4_-PEA were obtained from Epitech Group SpA (Saccolongo, Padova, Italy). *N*-heptadecanoyl-ethanolamine was purchased from Cayman Chemical (Cabru, Arcore, Italy). All solutions used for *in vivo* infusions were prepared using non-pyrogenic saline (0.9% wt/vol NaCl; Baxter Healthcare Ltd., Thetford, Norfolk, UK).

### Synthesis of [^13^C]_4_-PEA and preparation of an ultramicronized formulation

In order to limit interference from endogenous PEA and improve sensitivity and selectivity of the analytical method, ^13^C-labeled PEA was used. [^13^C]_4_-PEA was prepared from palmitic acid-1,2,3,4-13C4, 99 atom % ^13^C. Palmitic acid-^13^C_4_ (520 mg) was dissolved in 20 ml dry methanol containing 0.05 ml methanesulfonic acid. The resulting solution was refluxed under a dry nitrogen atmosphere for 2 h and then evaporated under vacuum. 1.25 g of ethanolamine was added and the resulting oily mixture warmed at 120°C in an oil bath for 4 h under a nitrogen atmosphere. The mixture was then cooled to room temperature and partitioned using ethyl acetate and water. The aqueous phase was discarded and the organic phase evaporated under vacuum. The residue thus obtained was crystallized from isopropanol, recovered by filtration and vacuum-dried. The final yield of pure naïve [^13^C]_4_-PEA was 480 mg, with a particle size range of 100–700 μm (Skaper et al., 2014).

[^13^C]_4_-PEA-um was produced by processing naïve [^13^C]_4_-PEA in a pilot spiral jet-mill micronizer (PILOTMILL-2, FPS Srl, Como, Italy), with pressurized nitrogen at 12 bar as carrier. Particle size distribution was assessed with a Laser Diffraction Particle Size Analyzer Mastersizer 3000 (Malvern Instruments Ltd, Malvern, UK): Dv 10 = 1.03 μm; Dv 50 = 2.52 μm; Dv 90 = 4.73 μm. Ten percent of the particles were smaller than 1.03 μm, 50% smaller than 2.52 μm, and 90% smaller than 4.73 μm; overall particle size range was 0.8–6 μm (Impellizzeri et al., 2014; Skaper et al., 2014).

### Animals

The study was carried out using Sprague–Dawley male rats (200–230 g, Envigo, RMS S.r.l., Udine, Italy). Food and water were available *ad libitum*. Experiments were performed in accordance with Italian Ministry of Health (art. 31, D.L. 26/2014) guidelines for the care and use of laboratory animals and EEC regulations (O.J. of E.C. L 358/1 12/18/1986). The University of Messina Review Board for the care of animals approved the study.

### Treatments

Treatment groups were arranged to measure the time-dependence of plasma levels of [^13^C]_4_-PEA in healthy rats receiving by oral gavage a single dose (30 mg/kg) of either [^13^C]_4_-PEA-um or naïve [^13^C]_4_-PEA dissolved in vehicle (1.5% carboxymethylcellulose wt/vol in saline). Parallel groups of rats so treated were concurrently subjected to a model of acute inflammation induced by intraplantar injection of carrageenan (CAR, 0.1 ml of a 1% suspension in 0.85% saline) into the right hind paw as previously described (Salvemini et al., 1996; Impellizzeri et al., 2016). Tissue (paw, spinal cord, brain) levels of [^13^C]_4_-PEA were also assessed in healthy and CAR-injected rats administered [^13^C]_4_-PEA-um, as follows:

- healthy rats receiving naïve [^13^C]_4_-PEA (*N* = 20);
- healthy rats receiving [^13^C]_4_-PEA-um (*N* = 20);
- CAR rats receiving naïve [^13^C]_4_-PEA, concurrently with CAR injection (*N* = 30);
- CAR rats receiving [^13^C]_4_-PEA-um, concurrently with CAR injection (*N* = 30).

Early absorption times were considered the most relevant for unmasking differences due to particle size, and healthy rats were sacrificed by anesthetic (isoflurane) overdose 5, 15, 30, and 60 min after [^13^C]_4_-PEA administration. Based on the time-course of CAR-associated inflammatory and hyperalgesic responses, two additional time points were investigated in the CAR-injected rats, i.e., 180 and 360 min. Five rats from each treatment group were sacrificed at each time point. Blood (from naïve [^13^C]_4_-PEA-treated rats, *N* = 20), as well as paw, spinal cord and whole brain collected at sacrifice were immediately frozen in liquid nitrogen and stored at −70°C for later [^13^C]_4_-PEA analysis.

Additional CAR-injected rats were used for assessing the effects of PEA-um. They received a single oral dose of PEA-um (10 mg/kg) dissolved as above and were randomly allocated to the following groups:

- CAR + saline: subjected to CAR-induced paw edema (*N* = 5);
- CAR + PEA-um: same as the CAR + saline group, but PEA-um was administered concurrently with CAR injection (*N* = 5).
- Sham-operated rats: the same surgical procedures as the CAR group, except that saline was administered instead of CAR (*N* = 5).

The dose and the route of PEA-um administration were chosen based on previous studies (Conti et al., 2002; Impellizzeri et al., 2014). At the end of the experiment (6 h post-CAR), rats were sacrificed by anesthetic (isoflurane) overdose. Samples from the hind paw and spinal cord (L4-L6) were collected and either fixed in 10% neutral-buffered formalin and embedded in paraffin for both histological and immunohistochemical examination, or stored at −70°C for further analyses. The 6-h time point was based on previous studies showing that the acute phase of inflammatory response and hyperalgesia (0–6 h) is characterized by central sensitization-related responses (D'Agostino et al., 2007, 2009), in other words, the spinal changes we were interested in to investigate the anti-hyperalgesic effect of PEA-um.

Different doses of PEA-um (30 mg/kg dose for blood/tissue analysis and 10 mg/kg dose for pharmacological study were chosen for the following reasons. *In vivo* studies have shown the most pharmacologically efficacious effective dose of PEA to be 10 mg/kg Esposito and Cuzzocrea, 2013, which was effective whether given before or after CAR injection (Conti et al., 2002). Concerning tissue analysis, prior experience showed high inter-individual variability in plasma levels for a 10 mg/kg dose, especially when using native PEA. Also, published data on blood/tissue levels following PEA administration were performed with higher doses [e.g., 100 mg/kg, Artamonov et al., 2005; Vacondio et al., 2015]) and 30 mg/kg (Petrosino et al., 2016; Siracusa et al., 2017). Further, sensitivity of the LC-APCI-MS analysis was a concern.

### [^13^C]_4_-PEA measurement by liquid chromatography/atmospheric pressure chemical ionization/mass spectrometry (LC-APCI-MS)

Plasma sample collection was performed as previously described (Petrosino et al., 2016). The levels of [^13^C]_4_-PEA in rat plasma and tissues were measured using the protocol previously described for PEA (Bisogno et al., 1997; Di Marzo et al., 2001; Marsicano et al., 2002; Petrosino et al., 2016), except that *N*-heptadecanoyl-ethanolamine was added as internal standard instead of [^2^H]_4_-PEA. Briefly, plasma and tissues were homogenized in chloroform/methanol/50 mM Tris-HCl pH 7.4 (2:1:1 by vol.) containing 10 pmol of *N*-heptadecanoyl-ethanolamine. The lipid-containing organic phase was pre-purified by open-bed silica gel chromatography, and the fractions obtained by eluting with chloroform/methanol (90:10 by vol.) were analyzed by LC-APCI-MS using a Shimadzu high-performance liquid chromatography apparatus (LC-10ADVP) coupled to a Shimadzu (LCMS-2020) quadrupole MS via a Shimadzu APCI interface. LC-APCI-MS analysis of PEA was carried out in the selected ion monitoring mode, using *m/*z values of 314 and 304 (molecular ions +1 for the standard and [^13^C]_4_-PEA, respectively); retention times were 17 and 13 min, respectively. [^13^C]_4_-PEA levels were calculated on the basis of their area ratios with the internal standard signal areas to give the amounts in pmol/ml of volume or pmol/g of tissue.

### Assessment of paw edema

Paw edema was measured with a plethysmometer (Ugo Basile, Comerio, Varese, Italy) prior to CAR injection and every hour for 6 h. Edema was expressed as the increase in paw volume (ml) after CAR injection relative to the pre-injection value for all animals. Scores were expressed as paw volume difference (ml).

### Nociceptive tests

Hyperalgesic responses to heat were assessed using the Plantar Test (Hargreaves method, Ugo Basile) with a cutoff latency of 20 s to avoid tissue damage (Hargreaves et al., 1988). Rats were individually housed in Plexiglas compartments and allowed to habituate. A mobile unit consisting of a high-intensity projector bulb was positioned to deliver a thermal stimulus directly to an individual hind paw from beneath the chamber. The withdrawal latency period of injected paws was determined with an electronic clock circuit and thermocouple. Results were expressed as paw withdrawal latencies. Behavioral testing was done with the experimenter blinded to treatment conditions.

### Histological evaluation

Seven micrometer-thick sections stained with haematoxylin and eosin were examined by light microscopy coupled to an Imaging system (AxioVision, Zeiss, Milan, Italy) and scored by two investigators in a blinded fashion. The degree of inflammation was evaluated according to a score from 0 to 5, as previously described (Bang et al., 2009; Impellizzeri et al., 2014).

### Myeloperoxidase (MPO) activity

The activity of MPO (an enzyme released by neutrophils and used as a marker of neutrophil infiltration) was assessed as previously described (Cuzzocrea et al., 2007). The rate of absorbance was measured spectrophotometrically at 650 nm. MPO activity was determined as the capacity to degrade 1 mM of peroxide within 1 min at 37°C, and expressed as units per g of wet tissue.

### Staining of mast cells

Identification of mast cells was performed in paw edema sections by blue toluidine staining as described previously (Ahmad et al., 2012). Mast cell density was expressed as the number of mast cells per unit area of hind paw tissue.

### Determination of cytokine levels in paw exudates

Tumor necrosis factor-alpha (TNF-α), interleukin (IL)-1β and IL-6 released in the paw exudates were measured by ELISA (R&D systems, Minneapolis, MN) as described previously by Salvemini et al. (1996), and the results expressed as pg per paw normalized to the volume of exudate recovered from each paw.

### Western blot analysis of IκB-α, nuclear factor-kappaB (NF-κB), inducible nitric oxide synthase (iNOS), cyclooxygenase-2 (COX-2), and manganese superoxide dismutase (MnSOD)

Western blot analysis was performed as previously described (Cordaro et al., 2016). The following primary antibodies were used: anti-IκBα (1:500, Santa Cruz Biotechnology, DBA, Milan, Italy), anti-iNOS (1:1000, BD-transduction, DBA, Milan, Italy), anti-COX-2 (1:1000, Cell Signaling- DBA, Milan, Italy), anti-MnSOD (1:1000, Santa Cruz Biotechnology) and anti-NF-κB p65 (1:500; Cell Signaling) at 4°C overnight in 1 × phosphate-buffered saline (PBS)/5% (w/v)/non-fat dried milk/0.1% Tween-20. To control for equal loading of protein lysates, blots were also incubated with either an anti-β-actin antibody (1:5000; Santa Cruz Biotechnology) for the cytosolic fraction or an anti-lamin A/C antibody (1:5000; Sigma-Aldrich) for the nuclear fraction. Importantly, the blot was stripped with glycine 2% and reprobed several times to optimize detection of proteins and to visualize other proteins without the need for multiple gels and transfers. The signals were revealed with a chemiluminescence detection system reagent according to the manufacturer's instructions (Super Signal West Pico Chemiluminescent Substrate; Pierce). Relative expression of protein bands was quantified by densitometry with BIORAD ChemiDocTM XRS+software and standardized to β-actin or lamin A/C levels. Pictures of blot signals (8 bit/600 dpi resolution) were imported to analysis software (Image Quant TL, v2003). Western blot analyses are representative of three different gels made by dividing the number of samples obtained from five animals for each experimental group repeated three times on different days.

### Immunohistochemical localization of nitrotyrosine

Immunohistochemistry was performed as previously described (Paterniti et al., 2010). Paw and spinal cord slices were incubated overnight with anti-nitrotyrosine rabbit polyclonal antibody (1:200 in PBS, v/v, Millipore- DBA, Milan, Italy). Sections were rinsed with PBS and incubated with peroxidase-conjugated goat anti-rabbit IgG (1:2,000 Jackson Immuno Research, West Grove, PA, USA). Specific labeling was detected with a biotin-conjugated goat anti-rabbit IgG and avidin-biotin peroxidase complex (Vector Laboratories, Burlingame, CA, USA). To authenticate the binding specificity for different antibodies, duplicate slices were incubated with only primary or secondary antibody; no positive staining was observed in these sections. Slices were quantitatively evaluated for a variance in immunoreactivity by computer-assisted color imaging (Leica QWin V3, Cambridge, UK). The percentage area of immunoreactivity (determined by the number of positive pixels) was expressed as percent of total tissue area (red staining). Replicates for all experimental conditions and histochemical staining were acquired from each rat in each experimental group. All analyses were carried out by two observers blinded to the treatment.

### Statistical evaluation

All values in the figures and text are expressed as mean ± standard error of the mean (SEM). Tissue level analyses were performed using the generalized linear model (GLM). In order to compare the levels of different formulations at different times Tukey-Kramer post-hoc analysis was used. In experiments involving histology and immunohistochemistry, the images shown are illustrative of at least three experiments done on different days on tissue sections collected from all animals in each group. The results were analyzed by one-way ANOVA followed by Bonferroni's multiple comparison test. Data were analyzed using SAS v9.2 (SAS Institute, Cary, NC, USA). A *p*-value of < 0.05 was considered significant. Exact p values are reported, unless < 1 out of 10,000 (reported as *p* < 0.0001), 0.0001 being the lower limit for the statistical program.

## Results

### Plasma levels of [^13^C]_4_-PEA in healthy and car-injected rats

**Figure 1:**
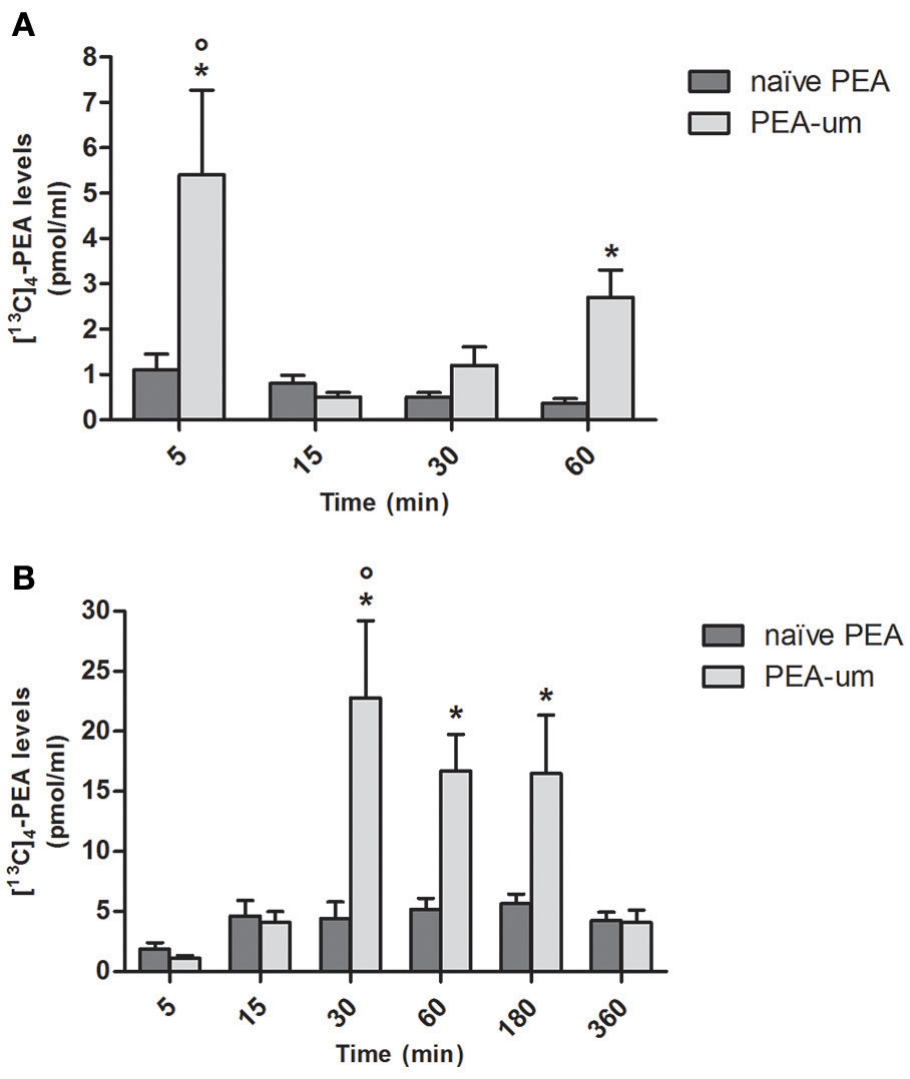
Effect of ultramicronization on plasma levels of [^13^C]_4_-PEA in healthy and CAR-injected rats. Levels of [^13^C]_4_-PEA in plasma of healthy **(A)** and CAR-injected **(B)** rats 5, 15, 30, 60, 180, and 360 min after oral administration of 30 mg/kg [^13^C]_4_-PEA-um or naïve [^13^C]_4_-PEA. Data are means ± SEM of five animals for each group. ^*^P < 0.05 vs. baseline; °*P* < 0.05 vs. naïve PEA.

To investigate whether particle size influences absorption of PEA, levels of [^13^C]_4_-PEA were first quantified in plasma of healthy rats 5, 15, 30, and 60 min after oral administration of the ultramicronized and naïve formulations (30 mg/kg). Mean plasma levels of [^13^C]_4_-PEA were significantly higher in rats receiving [^13^C]_4_-PEA-um than for naïve [^13^C]_4_-PEA (*p* = 0.0013) across all time points. In the former group, a significant peak concentration of [^13^C]_4_-PEA was found after 5 min (5.4 ± 1.87 pmol/ml, *p* < 0.0001), being 5 times higher than in rats administered the naïve formulation (1.1 ± 0.35 pmol/ml; *p* = 0.0078). There was a second, albeit lower peak of [^13^C]_4_-PEA at 60 min (2.7 ± 0.6 pmol/ml, *p* = 0.0006; Figure 1A) in [^13^C]_4_-PEA-um-treated rats. No significant peak plasma concentrations were found after oral administration of naïve [^13^C]_4_-PEA (Figure 1A).

The effect of particle size on plasma levels of orally administered [^13^C]_4_-PEA was next examined in CAR-injected animals. As for healthy rats, there was a significantly greater absorption for the ultramicronized as compared to the naïve formulation (*p* = 0.0013). An acute inflammatory state did not substantially alter absorption of naïve [^13^C]_4_-PEA, as plasma levels did not differ significantly from baseline at any time point. In contrast, [^13^C]_4_-PEA-um treatment resulted in higher levels of [^13^C]_4_-PEA across all time points in CAR-injected compared to healthy rats (*p* = 0.0046). Furthermore, marked increases in the absorption of orally administered [^13^C]_4_-PEA-um were observed at 30, 60, and 180 min compared to baseline (*p* < 0.0001 for each time, Figure 1B). Already 30 min after administration of [^13^C]_4_-PEA-um, plasma levels of [^13^C]_4_-PEA were 1.2 ± 0.41 pmol/ml and 22.8 ± 6.42 pmol/ml in healthy and CAR rats, respectively (*p* = 0.0345), this difference being maintained at 60 min (2.7 ± 0.6 pmol/ml and 16.7 ± 3.04 pmol/ml, respectively, *p* = 0.0191).

### Levels of [^13^C]_4_-PEA in tissues of healthy and car-injected rats

**Figure 2:**
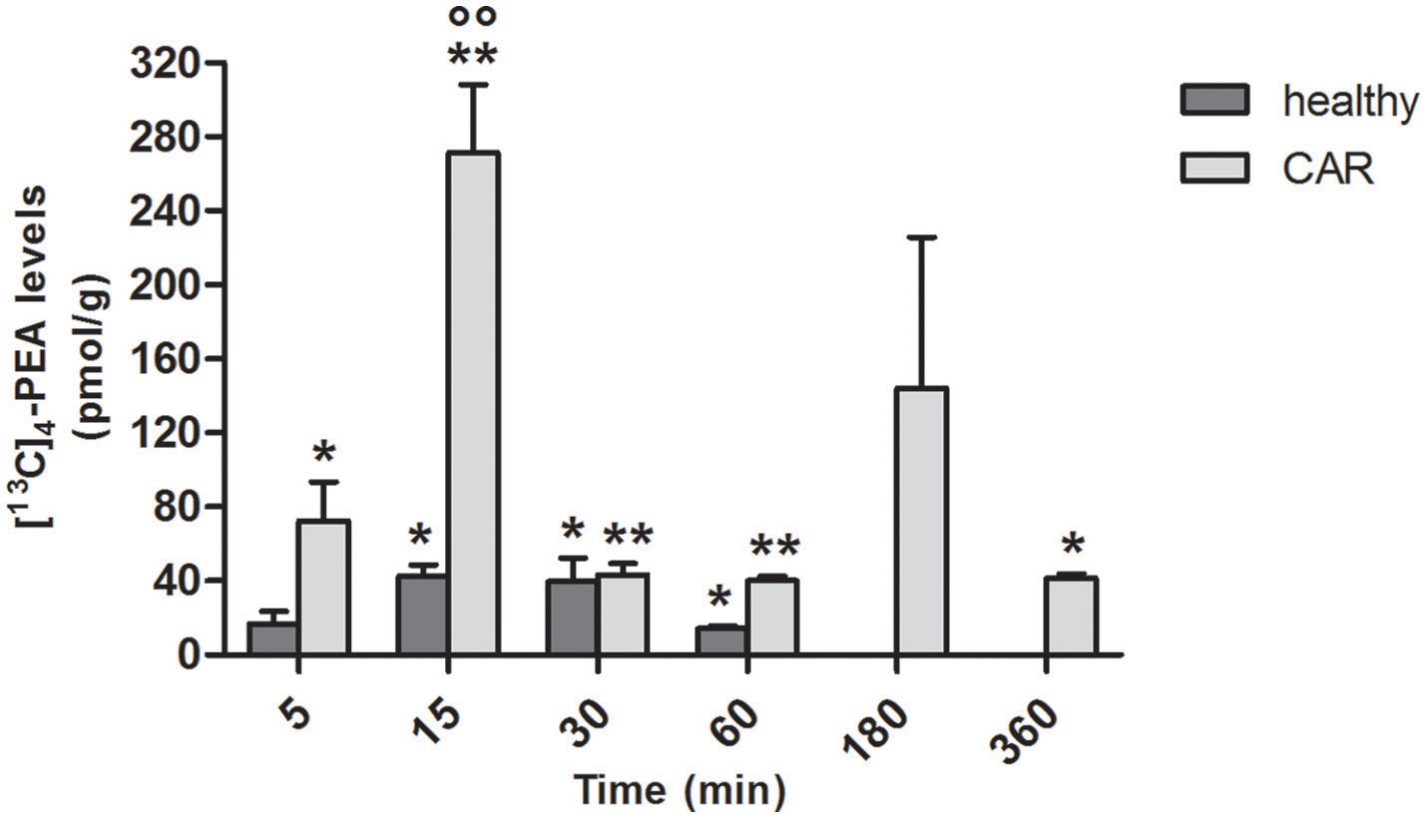
Time-course of [^13^C]_4_-PEA levels in paw tissue of healthy and CAR-injected rats following oral administration of [^13^C]_4_-PEA-um. Levels of [^13^C]_4_-PEA in the paw of healthy and CAR-injected rats were determined after oral administration of 30 mg/kg [^13^C]_4_-PEA-um. Data are means ± SEM of five animals for each group. ^*^P < 0.05 vs. baseline; ^**^P < 0.001 vs. baseline; °°*P* < 0.001 vs. healthy rats.

Since the absorption of PEA was higher following oral administration of ultramicronized compared to naïve [^13^C]_4_-PEA, both in healthy and (even more so) in CAR-injected rats, we next assessed tissue levels of [^13^C]_4_-PEA after a single oral dose of [^13^C]_4_-PEA-um. In paw tissue from healthy rats, levels significantly above baseline were observed at 15 min (*p* = 0.0070), 30 min (*p* = 0.0347) and 60 min (*p* = 0.0025). This was also the case for paw tissue collected from CAR-injected rats, being significantly higher than baseline at each time point, except *t* = 180 min (*p* = 0.0406, *p* = 0.0002, *p* = 0.0001, *p* < 0.0001, and *p* = 0.0031 at 5, 15, 30, 60, and 360 min, respectively; Figure 2). Notably, subplantar CAR injection resulted in a significantly higher distribution of [^13^C]_4_-PEA in the paw compared to healthy tissue across all time points (*p* = 0.0002). In particular, after 15 min the level of [^13^C]_4_-PEA in the paw of CAR rats was more than 6-fold higher compared to healthy rats (42.4 ± 6.39 pmol/g vs. 271.3 ± 36.93 pmol/g, respectively, *p* < 0.0001).

**Figure 3:**
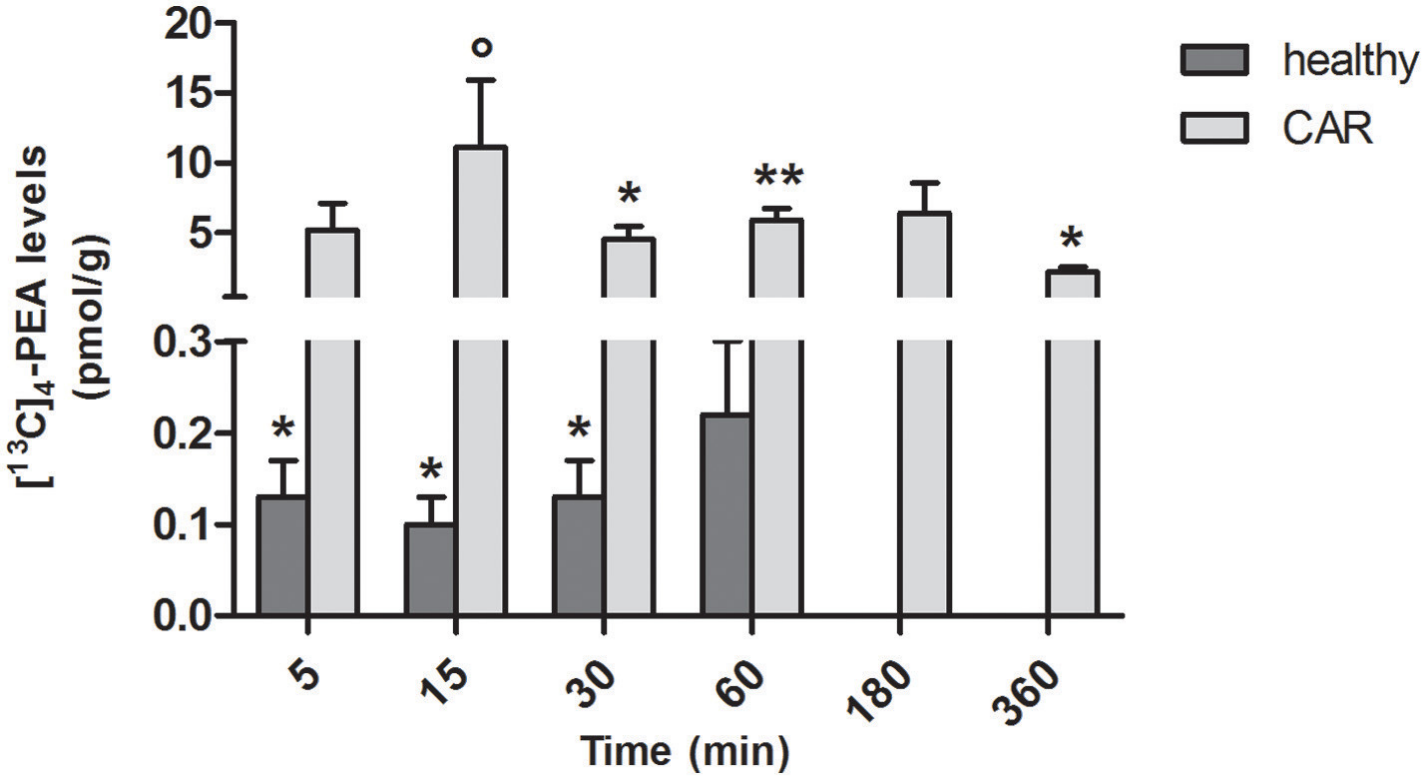
Time-course of [^13^C]_4_-PEA levels in spinal cord tissue of healthy and CAR-injected rats following oral administration of [^13^C]_4_-PEA-um. Levels of [^13^C]_4_-PEA in the spinal cord of healthy and CAR-injected rats were determined after oral administration of 30 mg/kg [^13^C]_4_-PEA-um. Data are means ± SEM of five animals for each group. ^*^P < 0.05 vs. baseline; ^**^P < 0.001 vs. baseline; °*P* < 0.05 vs. healthy rats.

In spinal cord of healthy rats, measurable amounts of [^13^C]_4_-PEA were found from 5 to 30 min (*p* = 0.0222, *p* = 0.0185, *p* = 0.0467 at 5, 15, 30, respectively; Figure 3). This was the case also for CAR-injected rats from 30 min (*p* = 0.0020, *p* = 0.0001, and *p* = 0.0030 at 30, 60, and 360 min, respectively). Interestingly, subplantar injection of CAR resulted in a significantly higher distribution of [^13^C]_4_-PEA also in spinal cord compared to healthy animals across all time points (*p* = 0.0004). Notably, 15 min after administration of [^13^C]_4_-PEA-um, [^13^C]_4_-PEA concentration in the spinal cord of CAR rats was 110-fold higher than in the healthy group (0.10 ± 0.03 pmol/g vs. 11.1 ± 4.79 pmol/g, respectively, *p* = 0.0396).

**Figure 4:**
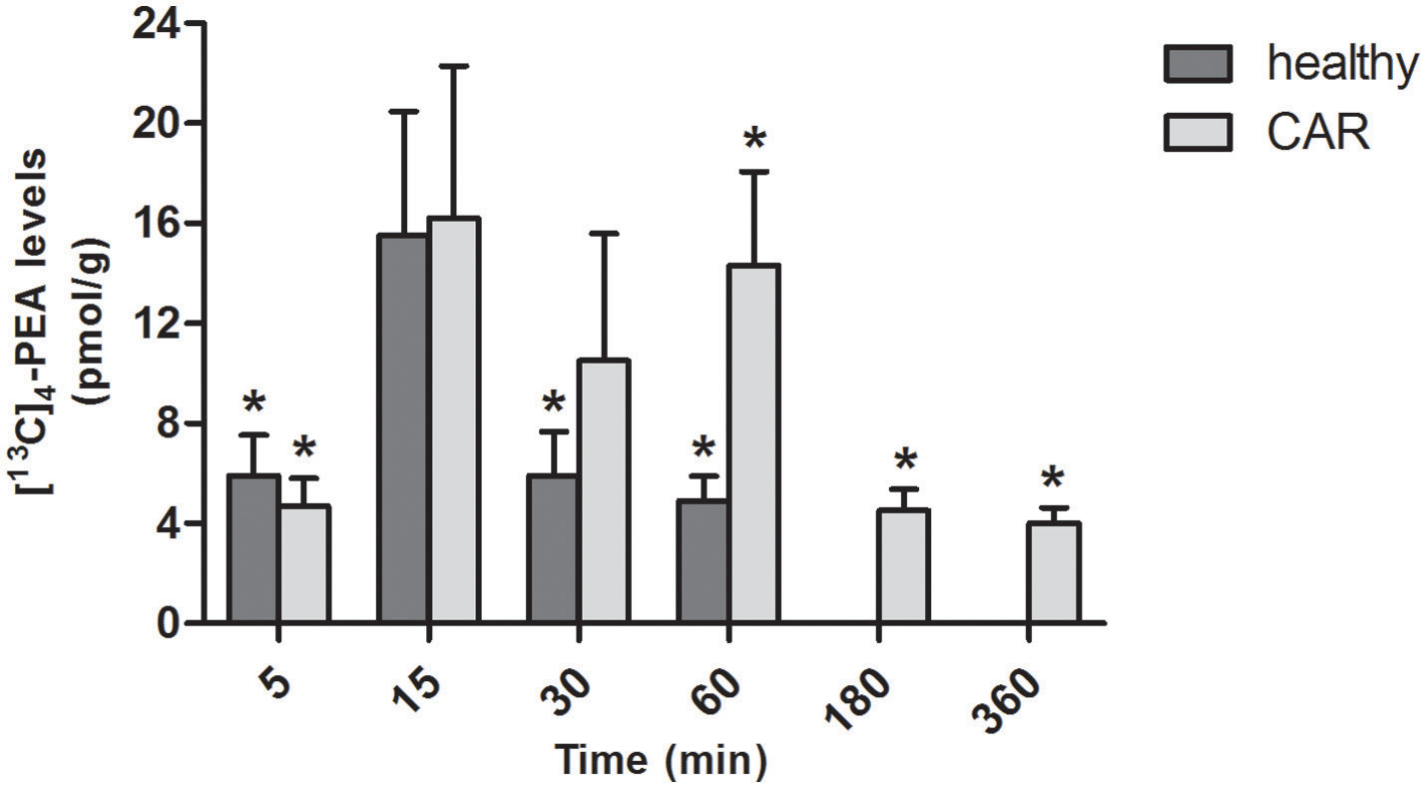
Time-course of [^13^C]_4_-PEA levels in brain of healthy and CAR-injected rats following oral administration of [^13^C]_4_-PEA-um. Levels of [^13^C]_4_-PEA in the brain of healthy and CAR-injected rats were determined after oral administration of 30 mg/kg [^13^C]_4_-PEA-um. Data are means ± SEM of five animals for each group. ^*^P < 0.05 vs. baseline.

In the brain of healthy rats, oral administration of [^13^C]_4_-PEA-um resulted in significantly increased levels of [^13^C]_4_-PEA compared to baseline at 5, 30, and 60 min (*p* = 0.0221, *p* = 0.0297, and *p* = 0.0074, respectively). Similar results were found in the brain of CAR-injected rats, the levels being significantly different from baseline at 5, 60, 180 and 360 min (*p* = 0.0129, *p* = 0.0043, *p* = 0.0068, *p* = 0.0083, respectively; Figure 4). Unlike paw and spinal cord, there were no consistent differences in the levels of [^13^C]_4_-PEA in brain between healthy rats and those receiving subplantar CAR injection at any time point.

### PEA-um counteracts car-induced rat paw edema and thermal hyperalgesia

**Figure 5:**
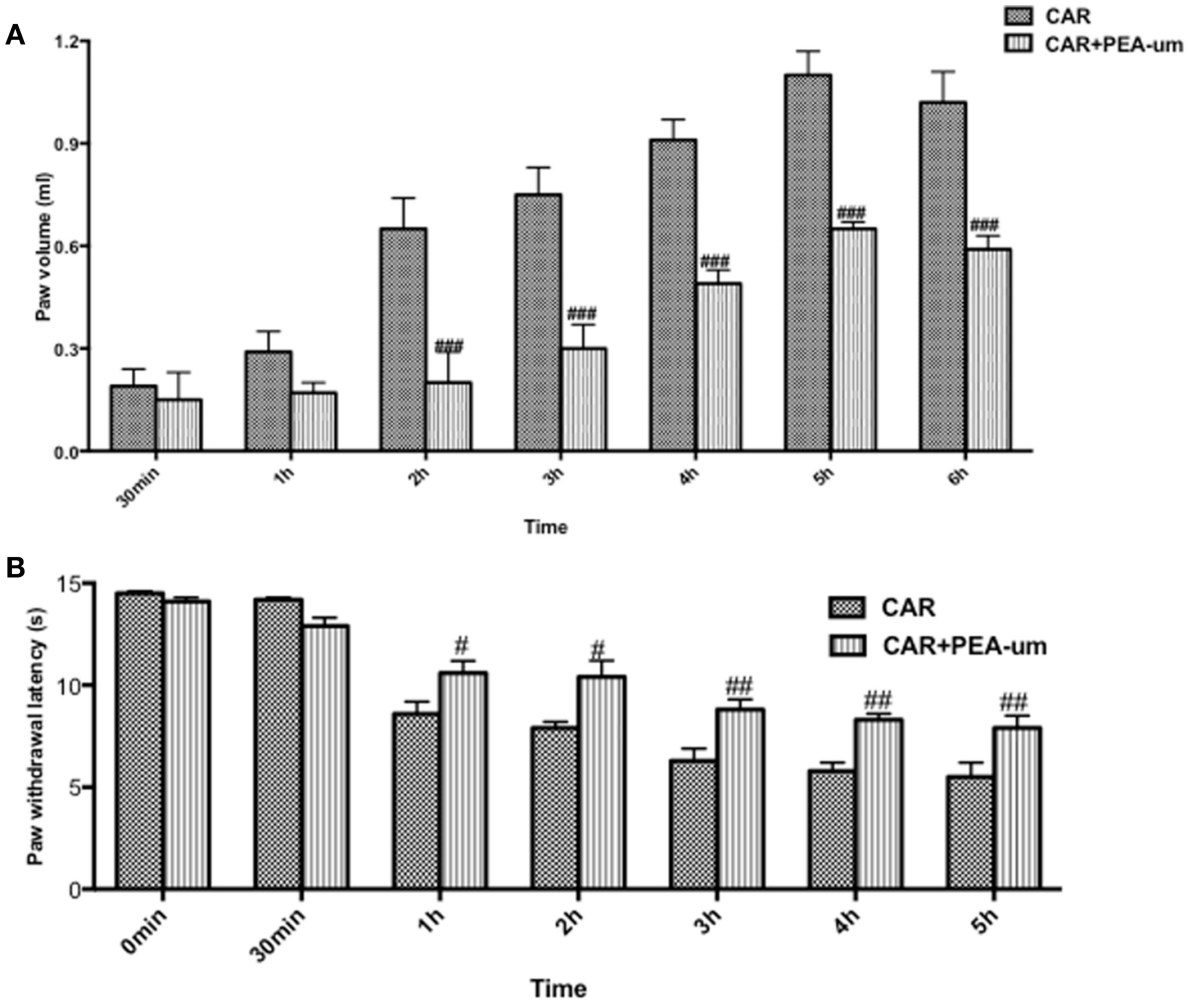
Effect of oral PEA-um on CAR-induced rat paw edema and thermal hyperalgesia. Paw edema **(A)** and thermal hyperalgesia **(B)** were assessed at the time points indicated after intraplantar injection of CAR into the rat hind paw. Oral administration of PEA-um (10 mg/kg) produced significant improvements in both scores. Values are means ± SEM ^#^*P* < 0.05, ^##^*P* < 0.01, and ^###^*P* < 0.001 vs. CAR.

Hyperalgesic responses involve both central and peripheral sensitization (Hargreaves et al., 1988; Urban and Gebhart, 1999). CAR intraplantar injection led to a time-dependent development of paw edema and thermal hyperalgesia, which peaked at 2–3 h and lasted for 6–8 h (Hargreaves et al., 1988; Salvemini et al., 1999). Oral administration of PEA-um (10 mg/kg) significantly reduced the development of paw edema beginning from the second h (Figure 5A) and thermal hyperalgesia from the first h (Figure 5B).

### PEA-um decreases car-induced histological damage and neutrophil infiltration in rat paw tissue

**Figure 6:**
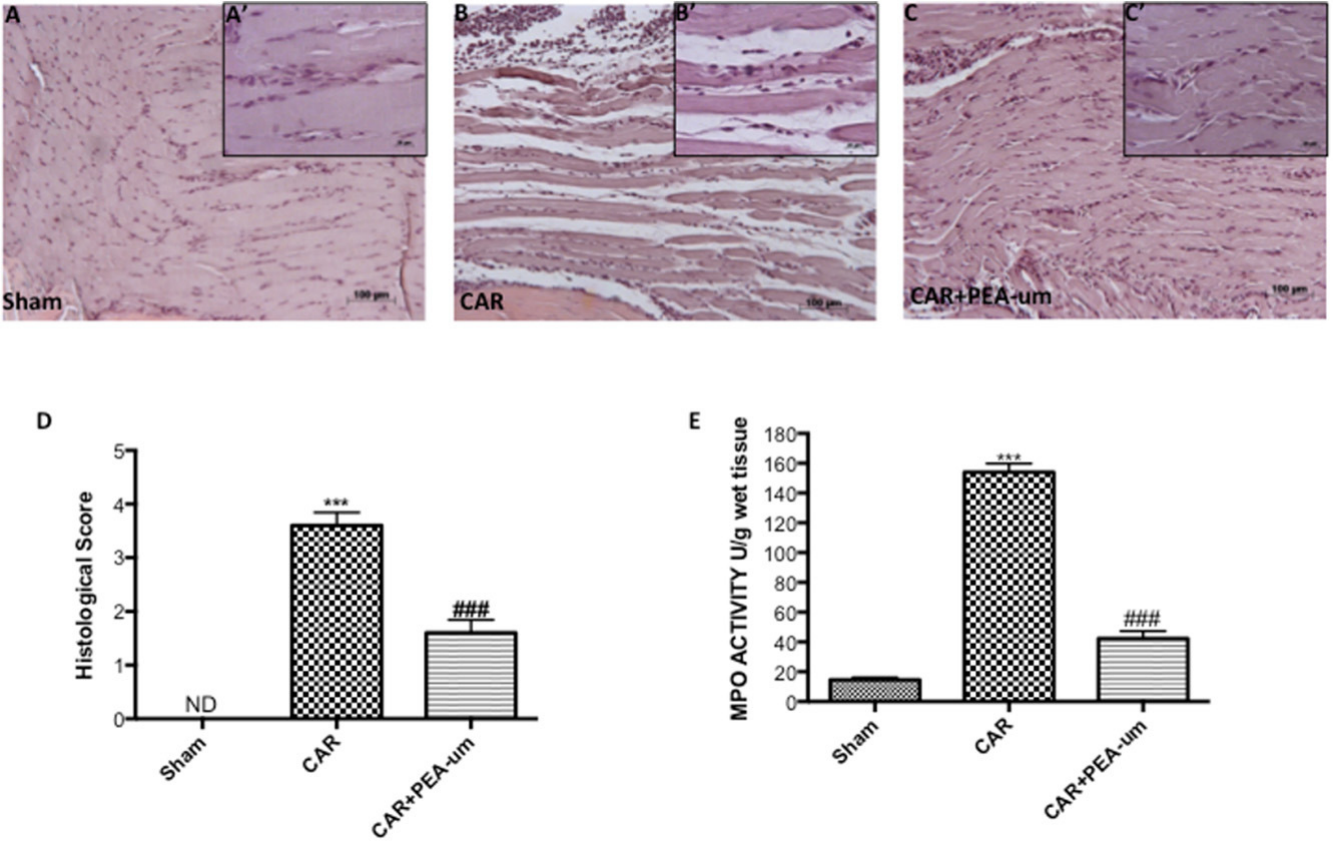
Effect of oral PEA-um on CAR-induced histological damage and neutrophil infiltration in paw tissue. Histological analysis was performed by hematoxylin/eosin staining. **(A)**, sham; **(B)**, intraplantar injection of CAR into the rat hind paw; **(C)**, CAR + PEA-um (10 mg/kg). Inserts **(A',B',C')** are higher-resolution images of the respective panels. **(D)**, histological scores. **(E)**, myeloperoxidase (MPO) activity in paw tissues from various treatment groups. Oral treatment with PEA-um produced significant improvements in both measurements. The figures are representative of at least three independent experiments for all animals from each group. Values are means ± SEM of five animals for each group. ^###^*P* < 0.001 vs. CAR; ^***^P < 0.001 vs. sham.

Tissue from sham-treated rats appeared normal (Figures 6A,A',D, histological score). In contrast, a marked accumulation of inflammatory cells (Figures 6B,B',D, histological score) was evident 6 h after CAR injection into the right hind paw. Oral treatment with PEA-um (10 mg/kg) significantly diminished this histological alteration (*p* < 0.0001), as well as inflammatory cell infiltration (Figures 6C,C',D, histological score). Progression of histological injury was associated with neutrophil infiltration as confirmed by an increase in MPO activity (Figure 6E). Oral treatment with PEA-um (10 mg/kg) significantly reduced MPO activity (*p* < 0.0001; Figure 6E).

### PEA-um decreases car-induced mast cell infiltration in rat paw tissue

**Figure 7:**
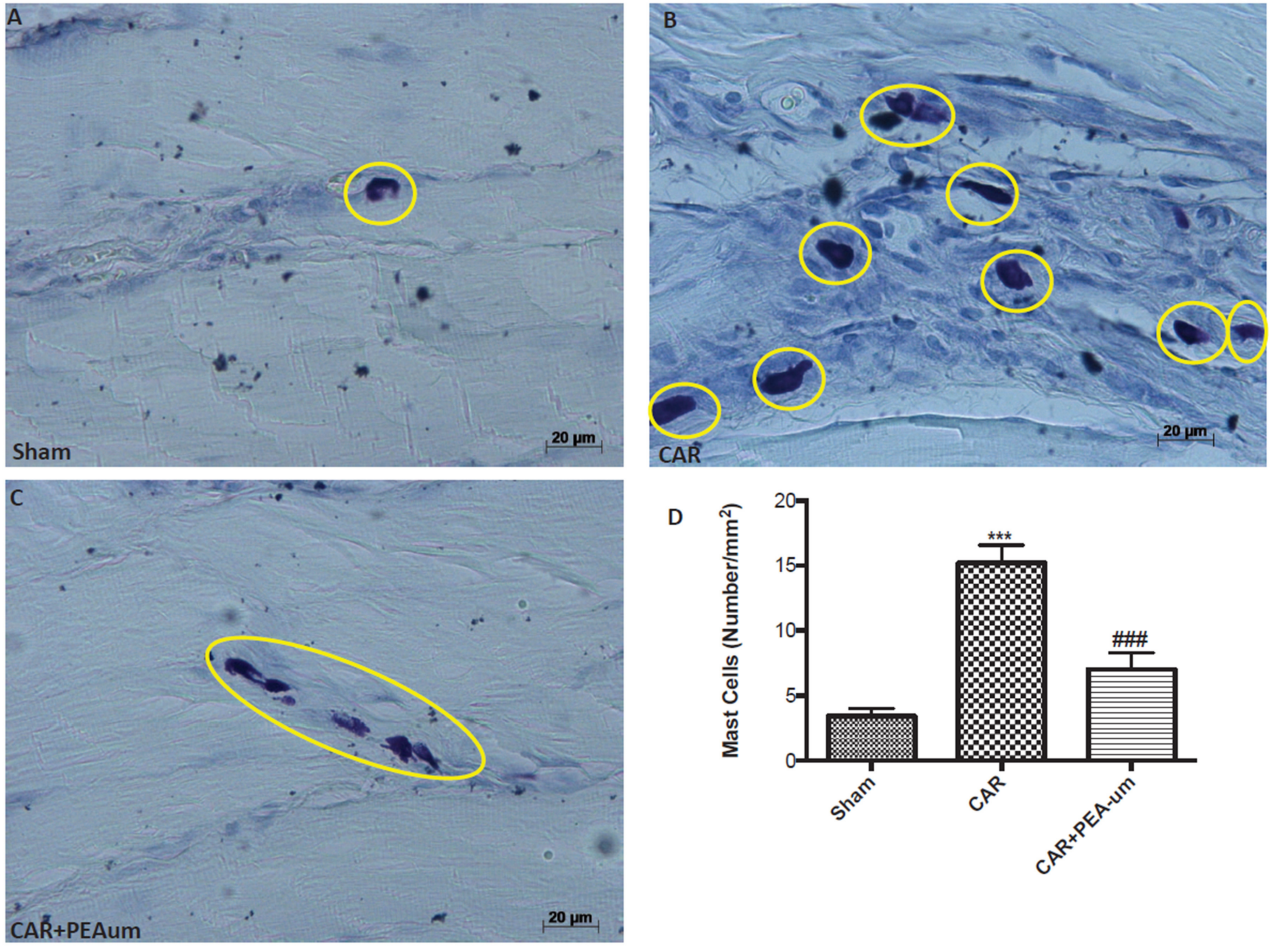
Effect of oral PEA-um on CAR-induced mast cell infiltration in rat paw tissue. **(A)**, sham; **(B)**, intraplantar injection of CAR into the rat hind paw; **(C)**, CAR + PEA-um (10 mg/kg); **(D)**, mast cell density. Oral treatment with PEA-um effected a significant decrease in the numerical density of toluidine blue-positive cells, as compared to the CAR group. The figures are representative of at least three independent experiments for all animals from each group. Values are means ± SEM of five animals for each group. ^###^*P* < 0.001 vs. CAR; ^***^P < 0.001 vs. sham.

CAR-injected rat paw tissue, stained with toluidine blue revealed a clear infiltration of mast cells (Figures 7B,D), as compared to sham animals (Figures 7A,D). Oral treatment with PEA-um (10 mg/kg) significantly decreased mast cell infiltration (*p* = 0.0003; Figures 7C,D).

### PEA-um reduces car-induced cytokine release, nitrotyrosine formation, and iNOS expression in rat paw tissue

**Figure 8:**
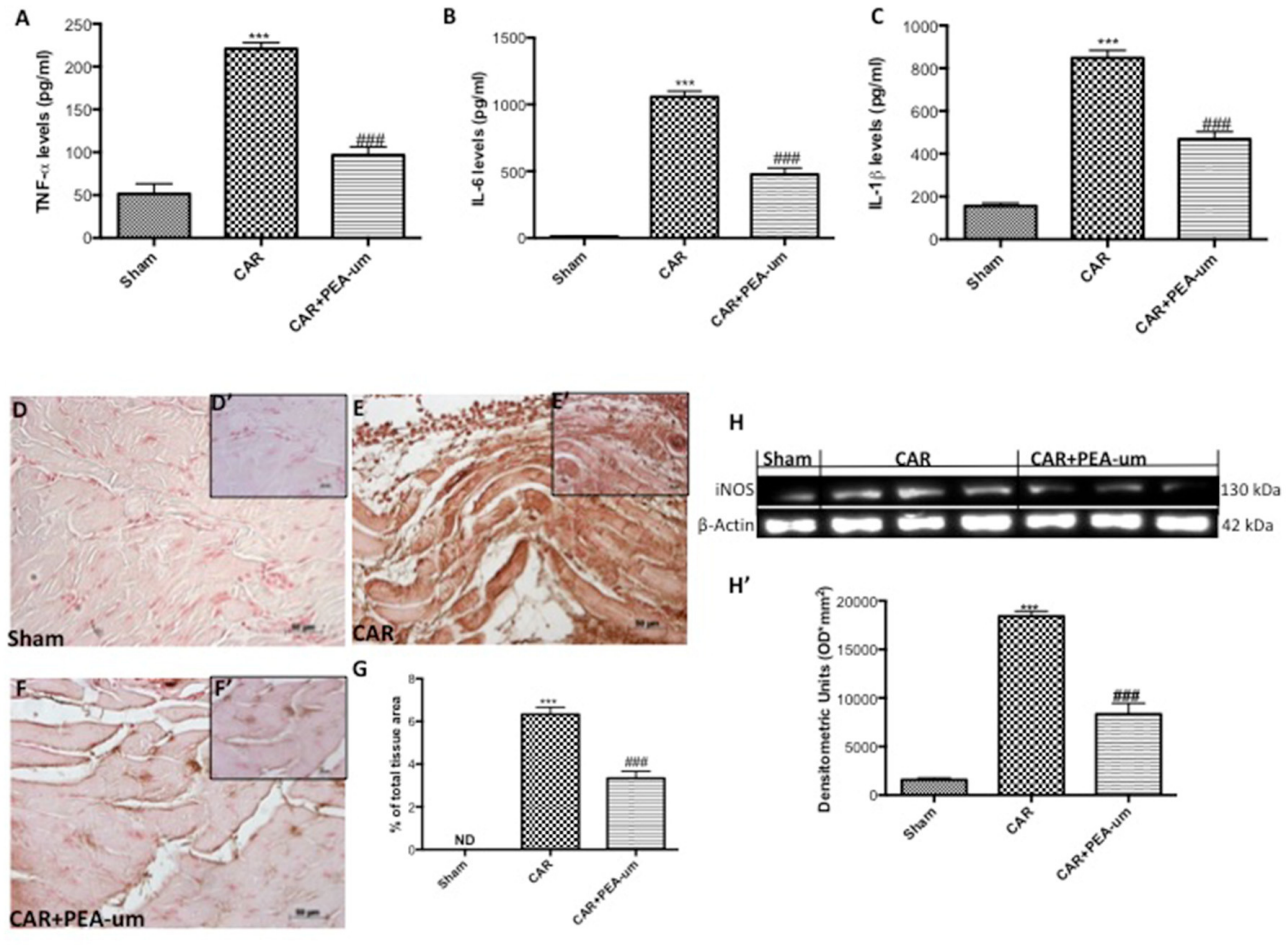
Effect of oral PEA-um on CAR-induced cytokine release, nitrotyrosine formation, and iNOS expression in rat paw tissue. A significant increase of TNF-α **(A)**, IL-6 **(B)**, and IL-1β **(C)** levels was detected in paw exudates after intraplantar injection of CAR into the rat hind paw. Oral treatment with PEA-um (10 mg/kg) significantly reduced levels of all three cytokines. Immunohistochemical staining for nitrotyrosine was positive in paw tissue sections from CAR-injected rats **(E,E',G)**, as compared to sham animals **(D,D',G)**. The intensity of nitrotyrosine staining was significantly reduced after oral treatment with PEA-um **(F,F',G)**. A significant increase of iNOS expression was observed in paw tissue from CAR-injected rats **(H,H')**, as compared to sham-treated rats **(H,H')**. Oral treatment with PEA-um significantly reduced iNOS expression **(H,H')**. A representative blot of lysates obtained from five animals for each group is shown, and densitometric analysis of all animals is reported. The figures are representative of at least three independent experiments and for all animals from each group. Values are means ± SEM of five animals for each group. ^###^*P* < 0.001 vs. CAR; ^***^P < 0.001 vs. sham.

The reduction of rat paw edema and thermal hyperalgesia by oral administration of PEA-um (10 mg/kg) was associated with a significant decrease in paw exudate content of pro-inflammatory and pro-nociceptive cytokines such as TNF-α (Figure 8A), IL-6 (Figure 8B) and IL-1β (Figure 8C), as compared to the CAR-injected group (*p* < 0.0001, *p* < 0.0001, *p* = 0.0001, respectively). Involvement of peroxynitrite in ROS-mediated nociception was evaluated by immunohistochemical detection of nitrated proteins (nitrotyrosine formation; Figures 8D–G). At the time of maximal inflammation and hyperalgesia (6 h), nitrotyrosine expression was clearly measurable in the inflamed paws and was associated with iNOS expression (Figures 8H,H', densitometric analysis). Formation of nitrated proteins and expression iNOS were significantly inhibited (*p* = 0.0001) by oral administration of PEA-um (10 mg/kg) (Figures 8F,G,H,H', densitometric analysis).

### PEA-um decreases car-induced COX-2 expression, IκB-α degradation and NF-κB p65 nuclear translocation in rat paw tissue

**Figure 9:**
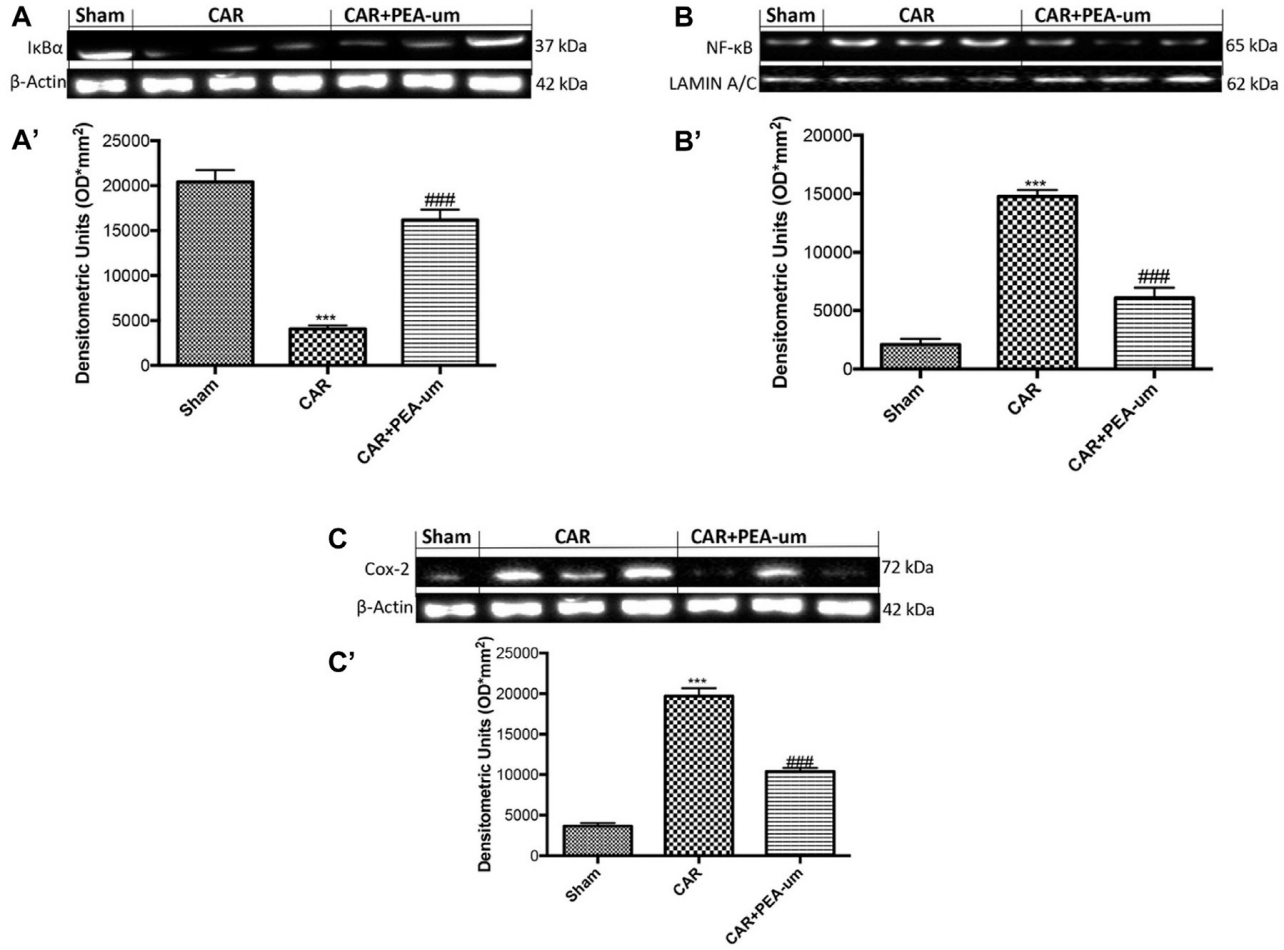
Effect of oral PEA-um on COX-2 expression, IκB-α degradation and NF-κB p65 nuclear translocation in rat paw tissue. The levels of IκB-α decreased in paw tissue homogenates from CAR-injected rats, as compared to sham-treated rats **(A,A')**. Oral treatment with PEA-um (10 mg/kg) significantly recovered IκB-α levels **(A,A')**. NF-κB p-65 translocation significantly increased in paw tissue homogenates from CAR-injected rats, as compared to sham-treated rats **(B,B')**, and PEA-um significantly decreased this effect. CAR-injected rats also displayed increased COX-2 expression as compared to sham-treated rats **(C,C')** which was significantly limited by oral PEA-um treatment. A representative blot of lysates obtained from five animals for each group is shown, and densitometric analysis of all animals is reported. Values are means ± SEM of five animals for each group. ^###^*P* < 0.001 vs. CAR; ^***^P < 0.001 vs. sham.

To better understand the molecular mechanism underlying the anti-inflammatory effects of PEA-um, IκB-α degradation and NF-κB p65 nuclear translocation were evaluated by Western blot analysis. The expression of IκB-α significantly decreased in rat paw tissue from CAR-injected rats, as compared to the sham-treated group (*p* < 0.0001; Figures 9A,A', densitometric analysis), and oral treatment with PEA-um (10 mg/kg) significantly limited CAR-induced IκB-α degradation (*p* = 0.0001). In contrast, translocation of the NF-κB subunit p65 increased in rat paw tissue from CAR-injected rats, when compared to sham rats (Figures 9B,B', densitometric analysis), with PEA-um (10 mg/kg) oral treatment significantly decreasing p65 translocation (*p* < 0.0001; Figures 9B,B', densitometric analysis). Given the COX-2 role in lipid degradation and subsequent production of leukotrienes and prostaglandins, we examined its expression by Western blot analysis 6 h after CAR injection and oral treatment with PEA-um. COX-2 expression significantly increased in rat paw tissue from CAR-injected rats as compared to the sham group (*p* < 0.0001 Figures 9C,C' densitometric analysis), and was significantly decreased with oral PEA-um (10 mg/kg) treatment (*p* = 0.0003; Figures 9C,C' densitometric analysis).

### PEA-um modulates car-induced MnSOD, COX-2, and iNOS expression in rat spinal cord

**Figure 10:**
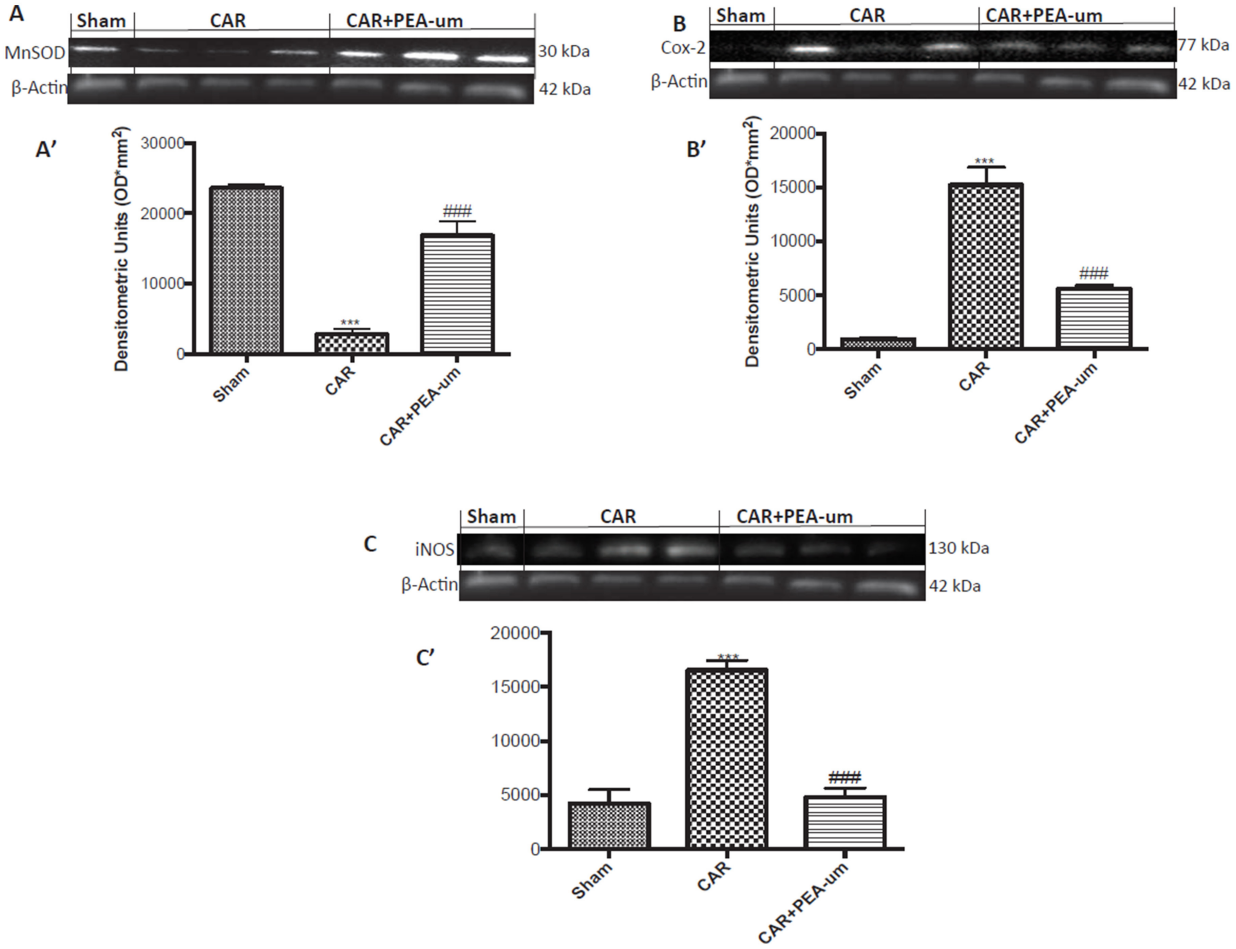
Effect of oral PEA-um on CAR-induced spinal MnSOD, COX-2 and iNOS expression. A significant decrease of MnSOD expression was observed in the spinal cord of CAR-injected rats, as compared to sham-treated rats **(A,A')**. Oral treatment with PEA-um (10 mg/kg) significantly restored MnSOD expression **(A,A')**. Increased COX-2 **(B,B')** and iNOS **(C,C')** expression was detected in the spinal cord from CAR-induced rats, as compared to sham-treated rats. Oral treatment with PEA-um reduced both parameters **(B,B',C,C')**. A representative blot of lysates obtained from five animals for each group is shown, and densitometric analysis of all animals is reported. Values are means ± SEM of five animals for each group. ^###^*P* < 0.001 vs. CAR; ^***^P < 0.001 vs. sham.

Central modulation of the nociceptive signal takes place in the lumbar tract of the spinal cord. Thus, we examined expression of the mitochondrial antioxidant MnSOD at L4/L6 to interrogate the involvement of central ROS (Esposito et al., 2016). MnSOD expression was significantly reduced in spinal cord 6 h after CAR injection, as compared to sham rats (*p* = 0.0002; Figures 10A,A' densitometric analysis). In contrast, oral treatment with PEA-um (10 mg/kg) significantly increased spinal MnSOD levels (*p* = 0.0002). As the acute phase of CAR-induced paw edema is characterized by central sensitization mediated primarily by prostanoids, we investigated spinal COX-2 and iNOS expression 6 h after CAR-injection and oral treatment with PEA-um. Indeed, COX-2 and iNOS expression were both significantly up-regulated (*p* < 0.0001 and *p* = 0.0001, respectively) in spinal cord from CAR-injected rats (Figures 10B,C,B',C', densitometric analysis, respectively) as compared to the sham-treated group. Oral treatment with PEA-um (10 mg/kg) significantly diminished spinal COX-2 (*p* = 0.0004; Figures 10B,B', densitometric analysis) and iNOS expression (*p* = 0.0001; Figures 10C,C', densitometric analysis).

### PEA-um modulates car-induced spinal IκB-α degradation and NF-κB p65 nuclear translocation

**Figure 11:**
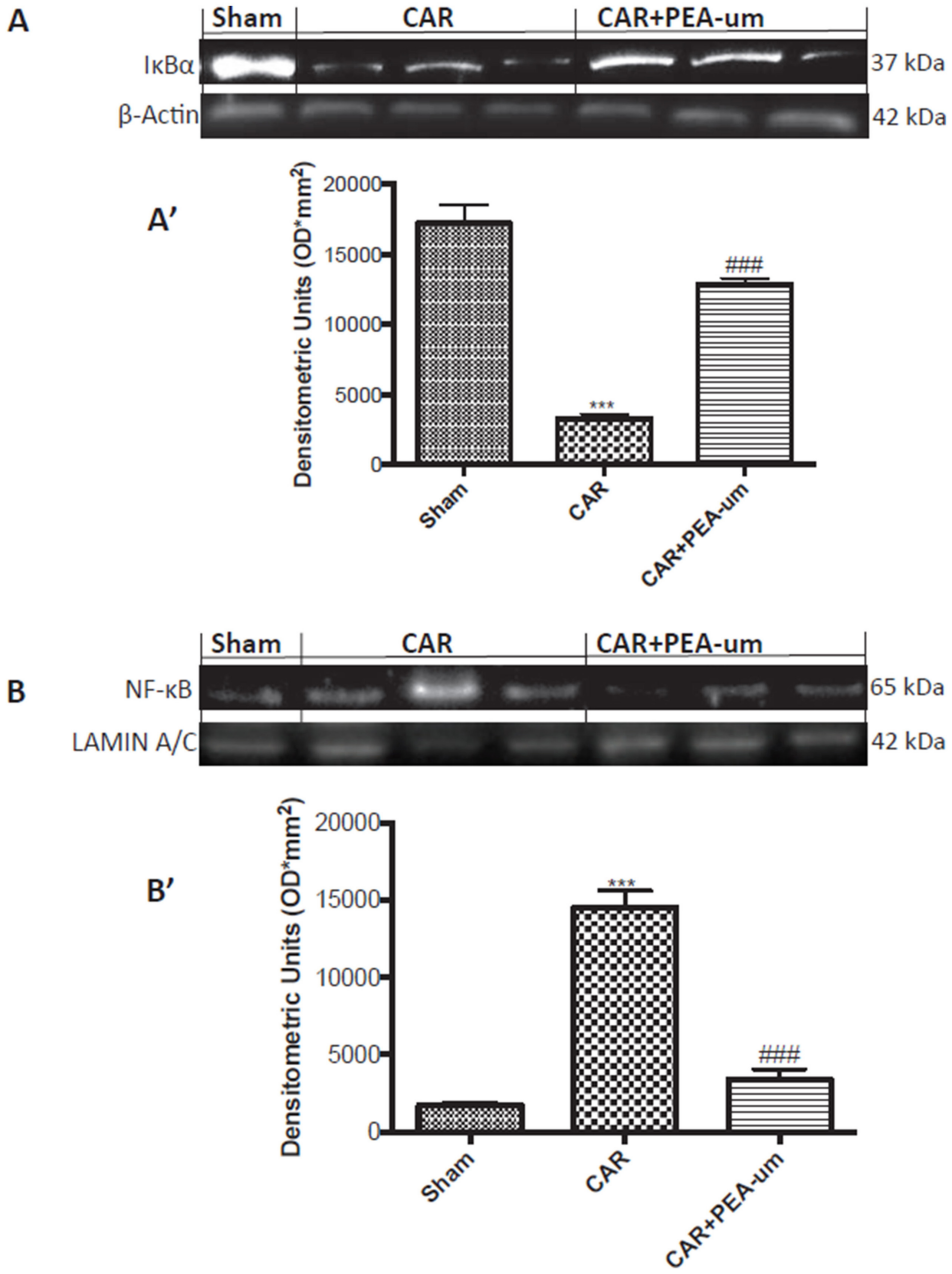
Effect of oral PEA-um on CAR-induced spinal IκB-α degradation and NF-κB p65 nuclear translocation. The levels of IκB-α significantly decreased in spinal cord tissue homogenates from CAR-injected rats, as compared to sham-treated rats **(A,A')**. Oral treatment with PEA-um (10 mg/kg) significantly recovered spinal IκB-α levels **(A,A')**. Further, levels of NF-κB p65 significantly increased in spinal cord tissue homogenates from CAR-injected rats **(B,B')**, and treatment with PEA-um significantly decreased NF-κB p-65 translocation. A representative blot of lysates obtained from five animals for each group is shown, and densitometric analysis of all animals is reported. Values are means ± SEM of five animals for each group. ^###^*P* < 0.001 vs. CAR; ^***^P < 0.001 vs. sham.

CAR injection provoked a statistically significant decrease of spinal IκB-α in the cytosolic fraction, as compared to the sham-treated group (*p* < 0.0001; Figures 11A,A', densitometric analysis). This effect was significantly prevented by oral treatment with PEA-um (10 mg/kg) (*p* = 0.0002; Figures 11A,A', densitometric analysis). Additionally, at the L4/L6 level, nuclear translocation of the NF-κB p65 subunit was significantly increased in the nuclear fraction 6 h after CAR injection, as compared to sham-treated rats (*p* < 0.0001; Figures 11B,B', densitometric analysis), and oral treatment with PEA-um (10 mg/kg) significantly reduced this effect (*p* < 0.0001).

## Discussion

An expanding body of literature surrounding PEA points to its promise in the application of a naturally-occurring molecule with anti-inflammatory and pain-relieving properties in the treatment of such conditions in both humans and companion animals (Re et al., 2007; Petrosino and Di Marzo, 2017). Oral delivery of drugs remains the most common route of administration, given its versatility, simplicity of administration and patient compliance. In the case of highly lipophilic agents like PEA with their low aqueous solubility and bioavailability, jet milling is often used to reduce large crystals down to the micron/submicron range and thereby enhance dissolution while increasing absorption and bioavailability (Chaumeil, 1998; Rasenack and Müller, 2004; Olusanmi et al., 2014). We therefore decided to first investigate plasma levels of PEA-um, and naïve PEA in healthy rats, exploiting a novel methodology based on the LC-MS measurement of synthetic and 99% isotopically pure [^13^C]_4_-PEA. This allows one to both limit bias from endogenous PEA and quantify mainly the exogenous compound and not its metabolites, although ethanolamine exchange cannot be excluded. It also avoids the pitfall relative to the recently reported PEA contamination in glass pipettes and the polyurethane foam used for their packaging (Angelini et al., 2017). Moreover, this methodology now permits one to determine, for the first time, the tissue levels of PEA after oral administration of a pharmacologically relevant dose.

Oral administration of [^13^C]_4_-PEA-um (30 mg/kg) to healthy rats resulted in [^13^C]_4_-PEA detectable in the bloodstream already after 5 min, with a peak plasma concentration of 5.4 ± 1.87 pmol/ml. In contrast, administration of naïve [^13^C]_4_-PEA did not yield a significant peak plasma concentration of [^13^C]_4_-PEA. Based on a basal value of around 9 pmol/ml PEA in rat plasma (Sharma et al., 2011; Wang et al., 2014), oral administration of PEA-um appears to rapidly elevate by some 50% circulating PEA levels. Vacondio et al. (2015) orally administered to rats a formulation of PEA (100 mg/kg suspended in corn oil and subjected to ultrasonication/vortexing) that reached a peak, 20-fold rise in plasma concentration after 15 min followed by a return to baseline within 2 h. In terms of particle size their formulation would likely be more similar to PEA-um than to naïve PEA. The discrepancy between (Vacondio et al., 2015) and our study (earlier and lower plasma peak following [^13^C]_4_-PEA-um oral administration) could reflect differences in: (i) bioavailablity related to particle size and vehicle; (ii) dosing; (iii) methodology to quantify plasma levels. Plasma levels of PEA have been measured also in human volunteers and beagle dogs after a single oral administration of PEA-m (~5 mg/kg) and PEA-um (30 mg/kg), respectively (Petrosino et al., 2016). In humans, PEA levels increased up to two-fold 2 h after administration (Petrosino et al., 2016), while in dogs, levels increased up to six-fold after 1–2 h. This difference could depend on species and doses used, but might relate to a relationship between absorption and particle size. Interestingly, after dosing PEA-um, a second plasma peak was found (Figure 1A), suggesting the possibility of enterohepatic recycling, although lymphatic transport cannot be ruled out.

PEA has an established anti-inflammatory profile and therapeutic utility in conditions characterized by hyper-activation of inflammatory and nociceptive pathways (Re et al., 2007; Esposito and Cuzzocrea, 2013; Petrosino and Di Marzo, 2017). Applying a classical model of acute inflammation (CAR-induced rat paw edema), levels of [^13^C]_4_-PEA were measured following a single oral dose (30 mg/kg) of ultramicronized or naïve [^13^C]_4_-PEA. These data confirmed the superior absorption behavior of the ultramicronized formulation, and more importantly, demonstrated significantly higher plasma levels of [^13^C]_4_-PEA in CAR rats compared to healthy animals dosed with [^13^C]_4_-PEA-um. Interestingly, following PEA-um administration, a rightward shift of the curve was observed in CAR-injected rats as compared to healthy animals. In particular, at 5 min post PEA-um administration the ratio of PEA levels in blood between healthy and CAR-injected animals was 4.9, in favor of the former group (Figures 1A,B). A feasible, albeit speculative, explanation might come from findings in paw tissues. As depicted in Figure 2, the ratio of PEA levels between healthy and CAR-injected rats at 5 min post treatment was 4.3 in favor of the latter. Although speculative, these differences in plasma and tissue distribution could reflect tissue needs for this natural bio-protector, with an early “flux” of PEA from blood to the CAR-injected paw (the injured/inflamed site), to establish an equilibrium between compartments (i.e., blood and the injury site) to serve their respective needs for PEA. Conceivably, and similar to the “on-demand” synthesis of endogenous PEA, there is “on-demand” distribution of exogenous PEA in CAR-injected rats, with PEA levels in blood rising by 5 min and then rapidly falling as PEA is re-directed to the paw.

In healthy rats orally administered [^13^C]_4_-PEA-um, [^13^C]_4_-PEA was detected in both spinal cord and brain—albeit consistently lower in the former—perhaps a consequence, in part, of the brain's higher perfusion rate compared to spinal cord (Marcus et al., 1977). Siracusa et al. (2017) reported a brain level of 21.68 ± 4.67 pmol/g [^13^C]_4_-PEA 15 min after oral administration of [^13^C]_4_-PEA-um (30 mg/kg) to healthy rats, in line with our determination of 16.20 ± 6.08 pmol/g. Further, following oral administration of [^13^C]_4_-PEA-um, levels of [^13^C]_4_-PEA in spinal cord of CAR rats were 26 to 110-fold higher than in healthy rats, perhaps due to transient changes in the blood-spinal cord-barrier that may occur secondary to CAR-induced peripheral inflammation (Xanthos et al., 2012). Indeed, increased penetration of certain drugs (e.g., morphine) into the spinal cord occurs in the CAR hind paw model (Lu et al., 2009). To some extent, levels of [^13^C]_4_-PEA in the paw of CAR rats were higher than in control, in line with the local increase of PEA that occurs at sites of inflammation (e.g., 2,4-dinitrofluorobenzene-mediated contact dermatitis in mice or skin of atopic dogs; Petrosino et al., 2010; Abramo et al., 2014). Initial studies carried out almost two decades ago based on intraperitoneal and oral administration of a particle size-uncharacterized radiolabeled PEA resulted in label in heart, lung, diaphragm, spleen, kidney, liver, testis, plasma, and brain (Zhukov, 1999; Artamonov et al., 2005). Because radioactivity was not specifically identified with the amide, one cannot exclude the possibility that the label detected actually reflected radioactive metabolites of PEA. More recently, Grillo et al. (2013) examined the distribution of PEA (10 mg/kg) in mouse tissues following its subcutaneous administration as a corn oil emulsion. In terms of particle size this PEA formation may be more similar to PEA-um than naïve PEA, having been ground and emulsified by vortexing/ultrasonification. PEA reached the blood, brain, retina and heart after both 24 and 48 h (Grillo et al., 2013). Although differences in route of administration, target species and PEA labeling/formulation render comparison with the present study impractical, the data of Grillo et al. (Grillo et al., 2013) encourage the view that micron-sized PEA administration favors prolonged blood absorption and tissue distribution. Moreover, evidence for passage of PEA through the blood-brain barrier also comes from studies carried out following intravenous administration of *N*-(16-(18)F-fluorohexadecanoyl)ethanolamine ((18)F-FHEA) as a positron emission tomography probe for imaging the activity of *N*-acylethanolamine hydrolyzing enzymes [namely the enzymes responsible of PEA metabolism (Iannotti et al., 2016; Petrosino and Di Marzo, 2017)] in the brain (Pandey et al., 2014).

Our findings on PEA-um blood absorption and tissue distribution, together with a prior study demonstrating superior oral efficacy of PEA-um compared to naïve PEA in the CAR-induced paw edema model (Impellizzeri et al., 2014), led us to investigate PEA-um effects in this model, focusing on aspects of the underlying molecular mechanisms. As pointed out earlier, the 6 h post-CAR time-point is considered optimal for evaluating central sensitization-related responses, that is, the spinal changes of interested to investigate the anti-hyperalgesic effect of PEA-um. Although the peak PEA concentration in paw tissue was reached before 6 h, this study does not allow to determine if there is a ‘lag time’ between the appearance of PEA in the tissue and its anti-hyperalgesic effect. Indeed, assuming a simple relationship between local tissue levels and the observed pharmacological effects may be unrealistic and, in every case, was not the focus of this investigation. That being said, we confirmed the reported effects of orally administered micron-sized formulations of PEA (10 mg/kg) on changes induced by CAR injection (Impellizzeri et al., 2014; Esposito et al., 2016; Petrosino et al., 2017), in terms of limiting paw edema, thermal hyperalgesia, neutrophilic infiltration (MPO activity), and tissue damage. PEA-um also counteracted the CAR-induced output of pro-inflammatory and pro-nociceptive mediators in paw tissues and exudates. Levels of IL-1β, TNF-α, and IL-6, and formation of nitrosylated proteins were significantly decreased in PEA-um-treated rats compared to the CAR-injected group, as described for PEA-m (Esposito et al., 2016) and PEA-um (Petrosino et al., 2017).

The early inflammatory response to CAR-induced edema results from release of mast cell histamine and serotonin (Di Rosa et al., 1971). Further, mast cell numbers increase in many inflammatory conditions (Kempuraj et al., 2004; Carvalho et al., 2006; Welker et al., 2008; Chang et al., 2009; Wang et al., 2009; Demir et al., 2013; Galdiero et al., 2017; Voisin et al., 2017). That PEA-um counteracted the CAR-induced increase in mast cell number is in accord with a recent report (Petrosino et al., 2017) and with the known ability of PEA to down-modulate mast cell behaviors (Aloe et al., 1993; De Filippis et al., 2011; Skaper et al., 2013), thereby reducing their number (De Filippis et al., 2011; Iuvone et al., 2016) and activation state (i.e., mediator release; Facci et al., 1995; Mazzari et al., 1996; Cerrato et al., 2010; Cantarella et al., 2011; Esposito et al., 2011; De Filippis et al., 2013; Donvito et al., 2015; Abramo et al., 2017).

Intraplantar injection of CAR decreases IκB-α expression and increases NF-κB p65 expression and activity (Wang et al., 2004), actions responsible for the increased expression of pro-inflammatory cytokines and activation of iNOS and COX-2 (Baeuerle and Baltimore, 1988; D'Agostino et al., 2007). Here, oral treatment with PEA-um significantly reduced COX-2 and iNOS expression, limited IκB-α degradation and decreased translocation of the NF-κB p65 subunit in paw tissue. One previous study examining the effect of oral PEA treatment on these nuclear factors in the CAR paw edema model showed similar results following administration of PEA-m or PEA co-micronized with the natural polyphenol polydatin (Esposito et al., 2016). Intracerebroventricular or spinal administration of PEA 30 min prior to CAR also prevented IκB-α degradation and NF-κB p65 nuclear translocation in the spinal cord (D'Agostino et al., 2007, 2009). In CAR-injected rats, spinal NF-κB was significantly decreased and IκBα increased following oral PEA-um treatment, effects consistent with the observed time-course of [^13^C]_4_-PEA appearance in spinal cord following oral administration of [^13^C]_4_-PEA-um under these conditions. Spinal mechanisms have not been previously studied following oral PEA-um administration, although similar results were reported for PEA-m and co-micronized PEA-polydatin (Esposito et al., 2016).

Peripheral inflammation enhances COX-2-mediated prostaglandin synthesis in the CNS, which contributes to nociception and hyperalgesia (Maihöfner et al., 2000; Ghilardi et al., 2005). Moreover, spinal cord iNOS is involved in CAR-induced inflammatory pain (Tao et al., 2003). Intraplantar CAR injection leads to a rapid induction of COX-2 in spinal cord and other CNS regions (Ichitani et al., 1997) and iNOS in spinal cord (Wang et al., 2014). Here, oral treatment with PEA-um reduced COX-2 and iNOS expression at the spinal level, as previously reported for co-micronized PEA-polydatin (Esposito et al., 2016). Analogous to the latter study, spinal MnSOD expression was increased by PEA-um treatment. Interestingly, MnSOD is suggested to play a role in controlling peroxynitrite formation through superoxide detoxification (Macmillan-Crow and Cruthirds, 2001) to counteract the development of hyperalgesia associated with acute inflammation (Muscoli et al., 2004; Wang et al., 2004). Thus, the effect on spinal MnSOD expression encourages a role for PEA-um treatment in counteracting peripheral as well as central sensitization. Moreover, inactivation of MnSOD contributes to the development of opiate-induced antinociceptive tolerance (Muscoli et al., 2007). That PEA-um counteracts the CAR-induced decrease of spinal MnSOD expression may shed new light not only on the anti-hyperalgesic effect of PEA-um on different kinds of pain but also on its ability to delay opiate tolerance (Di Cesare Mannelli et al., 2015). These latter data may also reflect the finding that oral PEA-um results in significant elevations in spinal cord over at least 6 h.

In conclusion, the present study shows that, in both healthy rats and those subjected to an acute peripheral inflammatory stimulus, orally administered PEA-um has a more favorable absorption profile compared to naïve PEA. In addition, these findings shed new light on spinal mechanisms involved in the anti-hyperalgesic effect of PEA-um in acute inflammatory pain.

## Author contributions

SC and EE: Conceived the study and participated in its design and interpretation of data; SP and VD: Were responsible for design and interpretation of data; SP, MC, VD, and SC: Drafted the manuscript; SP and VD: Edited the final manuscript; GM: Performed the synthesis of [^13^C]_4_-PEA and its ultra-micronization; AS and AP: Performed extraction and purification techniques; SP, RV, and FP: Performed LC-MS analysis; RS and DI: Performed *in vivo* experiments and Western blot analysis; RC and MC: Performed behavioral testing and histological analysis; CS: Performed the statistical analysis. All authors read and approved the final manuscript.

### Conflict of interest statement

SP, AM, and GM are employees of Epitech Group SpA. SC and VD are co-inventors on patent WO2013121449 A8 (Epitech Group SpA) which deals with compositions and methods for the modulation of amidases capable of hydrolyzing N-acylethanolamines useable in the therapy of inflammatory diseases. Moreover, SC is also a co-inventor with Epitech Group SpA on the following patents:

1. EP 2 821 083.
2. MI2014 A001495.
3. 102015000067344.

The other authors declare that the research was conducted in the absence of any commercial or financial relationships that could be construed as a potential conflict of interest.
