# Corrigendum: Oral ultramicronized palmitoylethanolamide: plasma and tissue levels and spinal antihyperalgesic effect

**DOI:** 10.3389/fphar.2024.1441203

**Published:** 2024-06-18

**Authors:** Stefania Petrosino, Marika Cordaro, Roberta Verde, Aniello Schiano Moriello, Gabriele Marcolongo, Carlo Schievano, Rosalba Siracusa, Fabiana Piscitelli, Alessio F. Peritore, Rosalia Crupi, Daniela Impellizzeri, Emanuela Esposito, Salvatore Cuzzocrea, Vincenzo Di Marzo

**Affiliations:** ^1^ Endocannabinoid Research Group, Institute of Biomolecular Chemistry, CNR, Napoli, Italy; ^2^ Epitech Group SpA, Padova, Italy; ^3^ Department of Chemical, Biological, Pharmaceutical and Environmental Science University of Messina, Messina, Italy; ^4^ Innovative Statistical Research SRL, Padova, Italy

**Keywords:** absorption, hyperalgesia, inflammation, micronization, palmitoylethanolamide

In the published article, there was a problem in Figure 9B as published. As a result, we have provided another representative image from our database, belonging to the antibody in question. The corrected Figure 9 and its caption appear below.

**FIGURE 9:**
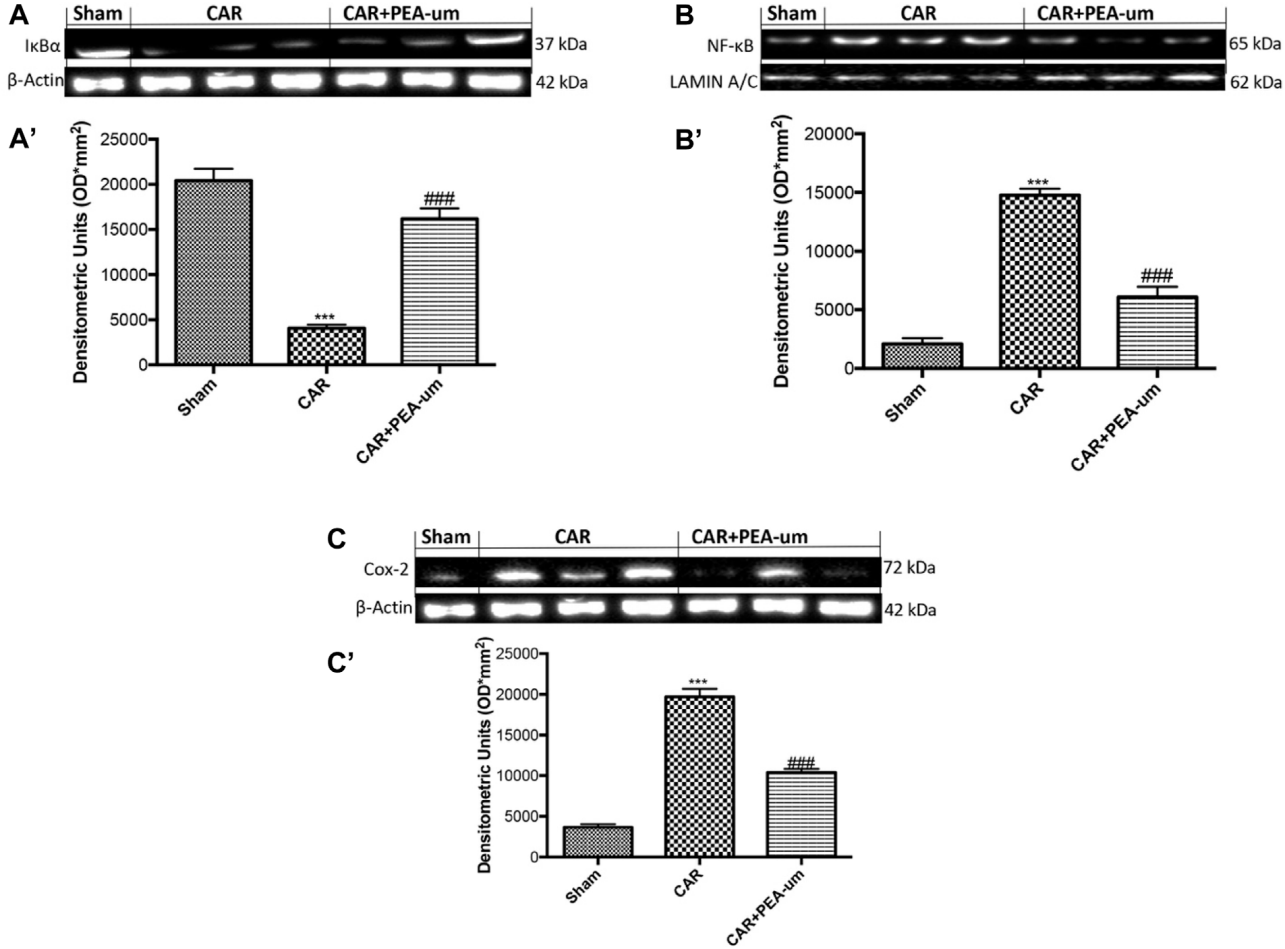
Effect of oral PEA-um on COX-2 expression, IκB-α degradation and NF-κB p65 nuclear translocation in rat paw tissue. The levels of IκB-α decreased in paw tissue homogenates from CAR-injected rats, as compared to sham-treated rats **(A,A′)**. Oral treatment with PEA-um (10 mg/kg) significantly recovered IκB-α levels **(A,A′)**. NF-κB p-65 translocation significantly increased in paw tissue homogenates from CAR-injected rats, as compared to sham-treated rats **(B,B′)**, and PEA-um significantly decreased this effect. CAR-injected rats also displayed increased COX-2 expression as compared to sham-treated rats **(C,C′)** which was significantly limited by oral PEA-um treatment. A representative blot of lysates obtained from five animals for each group is shown, and densitometric analysis of all animals is reported. Values are means ± SEM of five animals for each group. ^###^ *p* < 0.001 vs. CAR; ***p < 0.001 vs. sham.

The authors apologize for this error and state that this does not change the scientific conclusions of the article in any way. The original article has been updated.

